# Evaluation of field epidemiology training programs: a scoping review

**DOI:** 10.3389/fepid.2024.1376071

**Published:** 2024-06-27

**Authors:** Mohannad Al Nsour, Ghena Khasawneh, Yousef Khader, Haitham Bashier

**Affiliations:** ^1^ The Eastern Mediterranean Public Health Network, Amman, Jordan; ^2^ Department of Public Health, Jordan University of Science and Technology, Irbid, Jordan

**Keywords:** field epidemiology, training, evaluation, outbreak, surveillance

## Abstract

**Objectives:**

Field Epidemiology Training Programs (FETPs) are competency-based training programs that play a critical role in strengthening global health security and enhancing the epidemiological capabilities of public health professionals. This scoping review examined available published literature on the evaluations of FETPs globally.

**Methods:**

A literature review was conducted to evaluate FETPs globally. Keywords specific to the evaluation of FETPs were utilized to search the PubMed, Scopus, and Web Science databases. After applying the inclusion and exclusion criteria, 12 relevant studies from an initial pool of 60 were included in this study. Data extraction included key details, and a qualitative synthesis organized diverse findings using a narrative approach to draw appropriate conclusions and generate recommendations.

**Results:**

The review covered findings from 12 studies covering all three FETP modalities and spanning countries in various regions. Evaluations explored gained skills, engagement in FETP activities, and improvements in field epidemiological functions. Gained skills and knowledge, engagement in FETP activities, and improvements in field epidemiological functions were evident, with specific expectations for each FETP tier. Positive outcomes were consistent across studies, revealing improvements in surveillance activities, outbreak response, data management, and other system functions.

**Conclusion:**

This review confirmed the positive impact of FETPs on trainees and graduates, which emphasized competency enhancements across different modalities. Various strategies are recommended to improve the evaluation of FETPs. For effective evaluation, it is necessary to develop robust evaluation tools and establish standardized metrics to compare FETPs across regions or countries.

## Introduction

Field Epidemiology Training programs (FETPs) are competency-based training programs that play a crucial role in bolstering the national and regional health security infrastructure while also elevating the epidemiological capabilities of the public health workforce in health service organizations including the Ministry of Health or other national public health institutes (1, 2). FETP is considered one of the important activities of the US Centers for Disease Control and Prevention (CDC) that works on enhancing global health and well-being. It accomplishes this by providing comprehensive training, “learning by doing”, as 75% of the training program takes place in the field. FETP has three different modalities that include the 3-month basic program, which is also known as the frontline, the 1-year intermediate program, and the 2-year advanced program (2). The content of the program is tailored and customized depending on the circumstances of the country (1). FETPs train government public health professionals entrusted with national-level public health duties, particularly encompassing individuals such as physicians, public health officers, laboratory personnel, and veterinarians. Following the last four decades of investment, the FETP has achieved remarkable success, as there are now 90 FETPs serving over 200 countries worldwide (1).

The FETP aims to enhance the public health infrastructure of a country by strengthening and improving the health systems. This includes detecting, investigating, and responding effectively and quickly to public health incidents, establishing a strong surveillance system, building capacity in applied epidemiology, and guaranteeing that the decisions regarding public health are based on scientific data. The program emphasizes maximizing the trainees' field experience while minimizing classroom learning (1–3).

Globally, over 20,000 FETP graduates have been trained to identify and respond to a wide range of public health challenges and threats (1). Worldwide, more than 8,680 disease surveillance systems have been developed, more than 14,190 outbreaks or health events have been investigated, and over 11,250 poster presentations have been delivered at scientific conferences. In addition to that, over 3,710 peer-reviewed articles have been published (3).

There are several public health networks worldwide that work in partnership with the CDC to provide FETPs regionally or globally. These networks are instrumental in addressing the unique public health challenges faced by the region and enhancing regional cooperation in public health initiatives (1–6). These networks are critical as they improve public health by maximizing global efforts to respond to health crises. These programs focus on promoting global health security and advancing the skills of the public health workforce in field epidemiology (3). Thus being able to detect and respond to public health threats, which include humanitarian crises, natural disasters, and outbreaks.

Several evaluations for the FETPs have been conducted globally. These evaluations demonstrated improved skills and knowledge of trainees. However, they indicated that more efforts are needed to enhance the sustainability of the program (7–10). Thus, it is of paramount importance to conduct a comprehensive review of previous evaluations of FETPs and identify existing gaps. By examining the previous evaluations, valuable insights can be gained regarding the strengths and weaknesses of the programs. Thus helping to enhance the training curricula and ensuring that trainees receive the most relevant and up-to-date education, which bolsters their effectiveness in addressing health issues. Reviewing past evaluations and identifying FETPs becomes a linchpin in the quest to safeguard public health and advance the field of epidemiology. Hence, this this scoping review was conducted to answer the research question “What are the scope, nature, and outcomes of evaluations conducted on FFETPs?”. The study aimed to map the existing literature on the evaluation of FETPs and identify the scope, nature, and outcomes of these evaluations. Specifically, the study sought to aggregate and summarize key findings from previous FETP evaluations, focusing on their impact, assess the types of outcomes measured, the data collection methods used, and the involvement of different respondent groups, and identify the gaps and challenges reported in the implementation of FETPs.

## Methods

### Study design

A scoping review was conducted to comprehensively examine and map the landscape of evaluation studies assessing Field Epidemiology Training Programs (FETPs) globally. The aim was to gather, synthesize, and analyze existing evaluation reports that specifically assessed FETPs across various regions and contexts worldwide. This scoping review sought to identify the breadth and depth of research conducted on the evaluation of FETPs, including the methodologies used, key findings, and gaps in knowledge.

### Literature search

The literature search was limited to studies published between 2010 and 2023 because this ensures that the review includes the most current and relevant studies. Public health practices, training methodologies, and evaluation techniques have evolved significantly in the last decade, making studies from this period more applicable to current and future FETP implementations. The literature search was performed using three different databases, including PubMed, Scopus, Google Scholar, and Web of Science database. A search strategy was conducted focusing on multiple crucial keywords that included “Field Epidemiology Training Program”, “Frontline Field Epidemiology Training Program”, “Public Health Empowerment Program”, “Field Epidemiology and Laboratory Training Programs”, “Frontline field epidemiology”, “Basic field epidemiology”, “Field epidemiology service program”, “Epidemic Intelligence Service”, “Field epidemiology training programs for veterinarians”, “FETP”, “PHEP”, “PHEP-BFE”, or “Epidemiology intervention training”, combined with (AND) “Evaluation”, or “Assessment”. Boolean operators “AND” and “OR” were used to combine the key terms for widening and then narrowing the search strategy and reaching for the results to ensure retrieving wholesome literature specifically related to the topic. To search for the key terms in specific fields, the field tag [Title/Abstract] was used after the keywords. This would only limit the search to the specified (Title or Abstract) fields. Moreover, search results were limited to meet the inclusion and exclusion criteria mentioned as they set boundaries; the inclusion criteria focus on identifying the study population in a uniform, reliable, consistent, and objective manner (11).

### Inclusion and exclusion criteria

**Figure 1:**
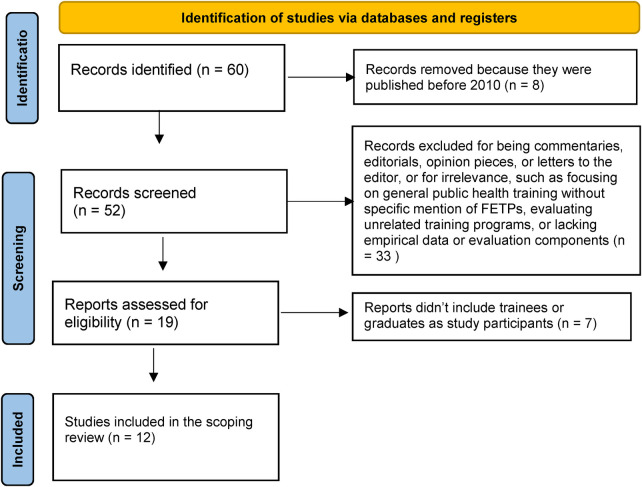
The PRISMA flow chart showing studies selection.

The inclusion criteria included studies that evaluated the FETP through qualitative, quantitative, or mixed method design and published between 2010 and 2023 in any language. Having access to the full text and reading it has helped in assessing if the study fits the inclusion criteria. Initially, 60 records were identified from three databases: PubMed, Scopus, and Web of Science. During the screening stage, the titles and abstracts of all 60 records were reviewed. Eight records were excluded for being published before 2010. An additional 33 records were excluded for being commentaries, editorials, opinion pieces, or letters to the editor, or for irrelevance, such as focusing on general public health training without specific mention of FETPs, evaluating unrelated training programs, or lacking empirical data or evaluation components (e.g., theoretical discussions or descriptive reports without outcome measures). Consequently, 19 full-text articles were assessed for eligibility. During this stage, seven full-text articles were excluded as they did not meet the inclusion criteria, specifically because they did not include trainees or graduates in their samples. Finally, 12 journal articles were included in this scoping review (7–9, 12–20). The flow chart (Figure 1) shows the study selection process.

### Data synthesis

A narrative approach was used to synthesize the data due to the differences in study design, FETP modality evaluated, evaluation outcome, data collection method and tools, and findings. This approach provides an organized structure identifying the key themes stated in each article. Following the title, abstract, and full-text review, the needed information from the selected papers was extracted into the Data Extraction Table, which is available in the “Results” section of this paper. Data extraction focused on the following data: country, region, year of evaluation, FETP modality, study design, evaluation outcomes, sampling, data collection and tools, main findings, gaps identified, and limitations.

## Results

### Studies characteristics

Out of 60 retrieved articles, 12 studies were included in this review. Table 1 presents the characteristics of the studies along with a summary of their findings. The evaluations covered the three different program modalities in various countries or regions, including Yemen (12), Ethiopia (13, 14), Guinea (15), Tanzania (16), United Kingdom (17), South Africa (18), Kenya (19), Ghana (20), India (9). Two studies evaluated the FETPs in selected countries in the EMR (7, 8). Five studies evaluated the frontline FETP (7, 13–15, 19), one study evaluated the intermediate FETP (16), and six studies evaluated the advanced FETP (8, 9, 12, 17, 18, 20). Ten studies were published between 2019 and 2023 (7, 8, 12–19), and two studies were published between 2011 and 2012 (9, 20). Of the 12 included studies, 9 evaluations were conducted utilizing the qualitative method, and 3 evaluations were conducted using the mixed methods.

**Table 1:** The characteristics of the studies along with a summary of their findings.

Author/year of publication	Country/Region	Year of evaluation	FETP modality	Study design	Evaluation outcomes	Sampling	Data collection and tools
Abduljalil et al. (12)	Yemen	2021	FETP-advanced	Mixed methods	Kirkpatrick's model levels 3 (behavior) and 4 (results)	5 FETPtechnical staff, 5 senior MoPHP policymakers who oversee the Y-FETP, 17 Program directors who hosted the FETP residents, 4 organizations that were employing the FETP graduates, and 43 FETP graduates	Desk review, FGDs, individual in-depth interviews, and an online survey
Findings: FETP helped 60% to 80% of graduates conduct outbreak investigations, surveillance analysis/evaluation, manage surveillance systems/projects, engage in public health communication (reports/presentation), and use basic statistical methods. The main gaps identified included: 1) FETP depends solely on donor support, which affects the sustainability of the program, and 2) FETP doesn't provide national coverage of all governorates							
Alsoukhni et al. (8)	Six countries in the EMR (Egypt, Iraq, Jordan, Pakistan, Yemen, Tunisia)	2023	FETP-frontline	Descriptive evaluation	Kirkpatrick's model levels 3 (behavior) and 4 (results)	A systematic random sample of 162 PHEP graduates and 8 directors/ technical advisers	Online survey
Findings: the majority of PHEP graduates reported that they are often involved in activities such as responding to disease outbreaks effectively (87.7%) and monitoring surveillance data collection (75.3%). High proportions of PHEP graduates rated their skills as good in performing most field epidemiology activities. The majority of graduates reported that the PHEP helped them much in conducting, reviewing, and monitoring surveillance data collection (92%), responding effectively to public health events and disease outbreaks (91.4%), and communicating information effectively with agency staff and with the local community (85.2%). Graduates from PHEP-Nutrition and PHEP-SPO programs reported significantly lower levels of perceived improvement in their ability to perform basic field epidemiology activities compared to graduates from the PHEP-BFE program							
Kebebew et al. (13)	Ethiopia	2019	FETP-frontline	Qualitative	FETP-Frontline's impact on surveillance and emergency management	41 interviews with key informants from FETP-Frontline implementing partners and 20 heads of district health offices	In-person key informant interviews
Findings: the program implementers shared positive perceptions towards the training program. The program has improved the knowledge and skills of district-level surveillance officers in a wide range of surveillance activities, including data quality improvement, early detection and prompt response to outbreaks, reporting, communication, etc. Districts with trained surveillance officers were perceived to perform better in surveillance activities compared to districts with untrained officers. Identified gaps included shortage of budget and human resources, poor mentorship, lack of career and professional development, shortage of medical equipment and supplies, poor internet access, political instability, mentors having limited time to devote to mentee support, unsatisfactory pay for officers, and staff turnover							
Collins et al. (15)	Guinea, West Africa	2018	FETP- frontline	Cross-sectional evaluation	Skills, as well as self-reported involvement in key activities related to data collection, analysis, and reporting	54 graduates of two cohorts, their current supervisors, and the director of one health facility	- Interviews and site visits. - Direct observation of data reports and surveillance tools at health facilities
Findings: the evaluation demonstrated a strongly positive perceived benefit of the FETP-Frontline training on the professional activities of graduates in support of surveillance and response functions, as well as the overall surveillance system. 2 months prior to the interview, 94% of graduates reported collecting data on notifiable diseases, 62% reported they had participated in an investigation, and 80% of graduates provided data analysis results back to the health facilities. About 76% of supervisors indicated an improvement in the completeness and timeliness of the reports, 49% indicated improvement in quality and analysis of the data, 97% indicated graduates involvement in analyzing case reports and data, 30% mentioned an improvement in the overall coordination and collaboration throughout the system, and 30% said the graduates were more motivated and engaged in surveillance activities. A total of 48 graduates (96%) said their analysis of the data enabled them to follow the trends of reportable disease, identify outbreaks early, initiate investigations, and use surveillance data to make recommendations to improve public health or surveillance procedures. About 96% of the health facility staff interviewed said there were positive changes in surveillance and response activities since the graduate's visit during their training. 60% reported that information sharing and case notifications had improved. Identified gaps included substantial gender imbalance in the first two cohorts and a tendency towards recruitment of more senior trainees approaching mandatory retirement age							
(Kebebew et al. (14)	Ethiopia	2017–2019	FETP-frontline	Cross-sectional study	Surveillance-related knowledge, skills, and performance among trained and untrained officers	150 district surveillance officers; 74 trained and 76 untrained	Structured questionnaire
Findings: FETP-frontline trained surveillance officers demonstrated better knowledge, skills, and performance in most surveillance activities compared to untrained officers. The completeness and timeliness of the weekly surveillance report were higher among the FETP-Frontline trained group than the untrained group. The trained officers were more likely to have produced epidemiologic bulletins (55% vs. 33%), conducted active surveillance six months before the survey (88% vs. 72%), provided surveillance training (88% vs. 65%), conducted strengths, weakness, opportunities, and threats (SWOT) analysis (55% vs. 17%), and utilized Microsoft Excel to manage surveillance data (87% vs. 47%). The availability of weekly reporting forms was not significantly different between the trained (97%) and untrained (93%) officers. The proportion of reports available, either in soft copy or hard copy, for 12 weeks before data collection was 75% for the trained group vs. 61% for untrained ones. The availability of surveillance summary and supportive supervision reports were also significantly better among the trained group. However, the availability of complete case-based forms and rumor logbooks were not significantly different among the groups							
(Wilson et al. (16)	Tanzania	2017–2020	FETP-intermediate	Descriptive evaluation	Knowledge and self-rated competency and trends in	53 FETP trainees	Knowledge tests and competency surveys
Findings: the program substantially improved trainee knowledge and competency and helped to improve local data quality and reporting. At the end of the program, most trainees reported overall improvements in the quality, timeliness, and completeness of surveillance data reported from their districts. Most trainees described positive changes at their worksites. Specifically, they felt more competent in performing audits and creating summaries of integrated disease surveillance and response (IDSR) data, analyzing data from surveys and outbreak investigations, and helping their colleagues to conduct these activities. Several trainees reported improvements in the timeliness and completeness of IDSR reports. Stakeholders remarked that the FETP Intermediate course had created a strong network of skilled epidemiologists to be recruited for investigating future outbreaks and strengthening data systems. Identified gaps included mentor availability during field assignments, limited time for data analysis practice, and difficulty balancing field assignments with work responsibilities. Some trainees reported challenges accessing routine data and survey participants for their field assignments because of limited flexibility in their work schedules and travel costs							
(Roka et al. (19)	Kenya	2017	FETP- frontline	Mixed methods	Kirkpatrick's model levels 3 (behavior) and 4 (results)	103 graduates (21 medical officers, 15 veterinary officers, 26 public health officers, 15 laboratory staff, 16 nursing staff, and 10 others), 12 supervisors, and 7 colleagues	Surveys, semi-structured interviews, data quality assessment (DQA) and data consistency assessment (DCA) scores, OTR percentages, and ratings of the training experience
Findings FETP- frontline proved overall data quality and on-time reporting (OTR) at the agency level but had minimal impact on data consistency between local, county, and national public health agencies. Participants reported that they acquired practical skills that improved data collation and analysis and OTR. The mean (DQA) score increased from 75.6% at baseline to 84.5% at 18 months postgraduation. There was an 11.4% improvement, but not statistically significant, in (DCA) scores between baseline and 18 months postgraduation. After training, it was noted that there is a significant increase in the mean knowledge/skill scores in each of the 8 assessed competencies. Most graduates, their supervisors, and their colleagues reported that the course had helped them to make scientifically based decisions and improved their overall capacity to deal with a spectrum of public health challenges, from calculating thresholds to responding to cholera cases. Additionally, they reported that the course helped them to become better leaders by improving their communication skills, enabling them to make more evidence-based decisions, and empowering them to show colleagues how to practically interact more critically with the data they generate at their agencies Limitations: first, the approach requires an assessment of participant learning needs and subsequent systematic training design. Second, participatory methods can be new and uncomfortable for individuals educated in formal or traditional styles, implying that programs with longer records and institutional memory may be hesitant to change. Third, systematically evaluating the short- and long-term effects of this approach beyond pretest and posttest questionnaires was challenging. It is possible that participants overrated or underrated their skills and knowledge when responding to survey items online. Many of the graduates did not respond to the repeated-measures surveys							
(Nsour et al. (7)	EMR (Saudi Arabia, Egypt, Jordan, Iraq, Morocco, Yemen, and Sudan)	2020	FETP-advanced	Descriptive	Kirkpatrick model for evaluation	166 FETP graduates and FETP 10 technical advisors	Online questionnaires
Findings: the FETP graduates in the EMR were well engaged in many field epidemiology activities including managing public health surveillance systems, surveillance data analysis, training public health professionals, and investigations on and response to outbreaks. The engagement of FETP graduates was the least in publishing research articles where only 28.3% reported that they are often engaged in writing scientific research articles. Moreover, less than half of the participants were often engaged in using epidemiologic methods to conduct studies that improve health program delivery, participate in public health research, and develop policy or strategy. Only four (40%) advisors reported that FETP graduates played a key role in regional-scale outbreaks. More than two-thirds of the FETP graduates rated their skills in conducting many field epidemiology activities as good. Five advisors (50%) reported that the data collection on reportable diseases has improved much in their countries since the establishment of FETP, and the rest reported that it has been somewhat improved. A total of seven (70%) technical advisors reported that the FETP has improved the investigations on and response to outbreaks in their countries to a large extent, and 30% reported that they are somewhat improved.							
(Dey et al. (17)	United Kingdom	2018	FETP-advanced	Mixed methods	Kirkpatrick's model levels 3 (behavior) and 4 (results)	14 consultant epidemiologists, 4 consultants in health protection/public health, 13 epidemiologists/epidemiological scientists, 4 information scientists, and 3 administrative staff members	Focus groups with supervisors and staff, Individual interviews with stakeholders, and an online survey for 28 graduates and 9 current fellows
Findings: the UK FETP appears to have substantively contributed to the capacity and quality of national field epidemiology provision. The perception was that these impacts have followed not only from training new staff but also indirectly from changing behaviors and maintaining skills within the wider field epidemiology workforce. Teams were confident in graduates’ capability when they returned to the service or took on new roles; graduates were seen to be knowledgeable and able to make decisions, work collaboratively with other disciplines and organizations, and have new and broader perspectives. FETP graduates continued to apply the skills they had developed following completion of the program and most (15/16 respondents) strongly agreed or agreed that completing the program had benefited their employing organization							
(Reddy et al. (18)	South Africa	2007–2016	(SAFETP)—advanced	Qualitative	Outputs of the trainees that included the core learning activities	98 residents	Outbreak investigations done, the number of abstracts presented, their evaluation for surveillance systems, the number of published manuscripts
Findings: SAFETP trainees and graduates have helped strengthen public health surveillance programs within the country. The trainees have contributed to the design, implementation, and evaluation of various surveillance systems at all levels of the health system. SAFETP trainees have made significant contributions in investigating and responding to numerous disease outbreaks and priority health conditions and control activities throughout the country that have been key inputs for local, provincial, and national public health decision-making. Over 45 scientific manuscripts have been published in peer-reviewed scientific journals by SAFETP residents. The identified gap included the program not attracting medical or veterinary graduates for enrollment							
(Bhatnagar et al. (9)	India	2001–2007	FETP- advanced	Qualitative	Assessment of the program's input, process, output, and outcome	80 trainees	Reviewing documents. Online survey
Findings -of the 80 students recruited during 2001–2007, 69 (86%) acquired seven core competencies. The faculty-to-student ratio ranged between 0.4 and 0.12 (expected: 0.25). Fieldwork led to the production of 158 scientific communications presented at international meetings and 29 manuscripts accepted in indexed peer-reviewed journals. The online survey showed that while most graduates acquired competencies, unmet needs persisted in laboratory sciences, data analysis tools, and faculty-to-student ratio. The results of the exit survey suggested that most graduates self-assessed themselves as proficient in all core competencies at the end of the program							
Wurapa et al. (20)	Ghana	2003–2011	FELTP- advanced	Qualitative	A matrix tool	37 residents	Needs assessment tools, residents’ outputs
Findings: there is ample evidence of improved public health surveillance and response as well as evidence-based decision-making taking place in the National Health Service following the joint evaluation of surveillance systems, disease dataset analyses, outbreak investigations, public health interventions with more regular reports, information sharing and periodic stakeholders’ public health seminars at all levels. The outputs of the residents have demonstrated the scientific rigor that has characterized the field investigations and dissertations that have been produced. The emphasis on scientific writing and communication has also been reflected in the oral and poster presentations that residents from the program have made in regional and global scientific conferences							

The samples included different respondents, as some evaluations included more than one group of respondents. Five studies included FETP graduates in the sample (7, 8, 12, 15, 19). Four studies included trainees/residents (9, 16, 18, 20). Two studies included the technical advisors (7, 8). Furthermore, two studies included the program directors (7, 12). Moreover, one study included trained and untrained surveillance officers (14). One study included the FETP graduates’ supervisors (15). One study included the technical staff (12). In addition, some studies included the policymakers (12), key informants from FETP implementing partners (13), heads of district health offices (13), a director of a health facility (15), epidemiologists, consultants in health protection, information scientists, and administrative staff (17).

Regarding data collection, surveys and questionnaires were used in eight studies (7–9, 12, 14, 16, 17, 19). Interviews were used in five studies (12, 13, 15, 17, 19). Also, in-depth and focus group discussions were used in two studies (12, 17). Some studies used desk review (12), site visits and supervision (15, 20), checking the papers published in peer-reviewed journals as an output of the program (8, 18, 20), reviewing the conference abstracts done (18, 20), observing the field reports for data collection (15, 20) and surveillance tools (15).

The included studies investigated various outcomes including gaining skills and improvement in knowledge (either self-rated or assessed by the advisors or supervisors) (7–9, 13–15, 19), involvement in FETP activities (8, 9, 18, 20), improvements in field epidemiological functions (8, 9, 15, 16, 19, 20), program gaps (9, 12, 13, 15, 16, 18), and other outcomes (8, 9, 16–19).

Kirkpatrick's model [level 3 (behavior) and level 4 (results)] was used in five studies to evaluate the outcomes of the FETP (7, 8, 12, 17, 19). Two studies evaluated the outcome by comparing the difference in performance among the trained and untrained people (13, 14). Two studies considered the acquired skills (14, 15) and two studies considered the learning activities (15, 18) for the evaluation outcomes. One study examined the impact on surveillance and emergency management (13). Another study evaluated the outcome by identifying the core components of the program by breaking it into input, process, output, and outcome (9). One study evaluated the outcome through a matrix tool (20). In addition, one study looked at the trends in disease surveillance and response (IDSR) data (16).

### Evaluation outcomes

#### Gained skills and knowledge

Seven studies provided insights and focused on the impact of FETPs on the perceived skills and knowledge of the trainees to address public health issues (7–9, 13–15, 19). The FETP was found to have a beneficial effect on the skills of the trainees regardless of whether the modality is frontline FETP (7, 13–15, 19) or advanced FETP (8, 9). Improvements in surveillance activities skills have been accentuated in the studies. The frontline FETP evaluation study in the EMR showed that numerous PHEP graduates assessed their proficiency positively in most field epidemiology activities (7). The frontline FETP evaluation study in Ethiopia (13) showed favorable perceptions by program implementers regarding the enhanced skills and knowledge in surveillance activities encompassing improvements in data quality, early outbreak detection, immediate response, reporting, and communication. In addition, India's advanced FETP evaluation study illustrated that the majority of the students self-assessed their competencies in the exit survey after they finished the program as proficient (9).

The studies that evaluated the frontline FETP in Ethiopia and Kenya showed that the skills, knowledge, and performance in surveillance activities were better trained when compared with untrained officers (13, 19). The advanced FETP evaluation study in Kenya illustrated that the majority of the graduates, supervisors, and colleagues stated that the training program contributed to enhancing their capability to be decision-makers based on scientific principles and strengthening their competence to deal with public health issues (19). The advanced FETP evaluation study in the EMR showed that two-thirds of the graduates assessed their skills as “good” in conducting field epidemiological activities (8).

Improvements in field epidemiology activities have been highlighted. In the frontline FETP evaluation study in the EMR, it was noted that PHEP had an impact on the capacity of 92% of the graduates, as they were able to conduct, review, and monitor surveillance data collection. Similarly, around 91.4% noted that PHEP favored its effectiveness in responding to disease outbreaks and public health issues (7). Also, another frontline evaluation study in Guinea illustrated that 30% of the supervisors mentioned that the graduates were more engaged in surveillance activities as improvements have been noticed in their collaboration and overall coordination throughout the system (15).

Several studies illustrated that an improvement was noticed in the communication skills of the graduates or trainees (7), and enhanced communication skills were noticed by the program implementers (13). Moreover, graduates reported that they have improved communication skills which had a positive impact on making them better leaders (19).

#### Involvement in FETP activities

Three studies provided insights regarding the involvement of the trainees and graduates in FETP activities (8, 18, 20). The advanced FETP evaluation in the EMR showed an increased participation of FETP residents or graduates in field epidemiology activities including investigating and responding to outbreaks, managing public health surveillance systems, training public health professionals, and surveillance data analysis (8). The South African FETP study showed that the residents addressed several outbreaks in the African region and they were involved in designing, implementing, and evaluating surveillance systems, thus, strengthening the public health surveillance system (18).

The advanced FETP evaluation in Ghana showed that the residents had scientific precision that has been shown in the dissertations and field investigations. Ghana FELTP (GFELTP) graduates were positioned as epidemiologists within disease control and public health programs at the national and subnational levels (20).

#### Improvements in field epidemiological functions

The five studies that assessed the impact of FETP on epidemiological functions showed improvements in field epidemiological functions (8, 15, 16, 19, 20). The Intermediate FETP evaluation in Tanzania showed marked improvements in the data quality, reporting, and outbreak investigations. The trainees reported enhancements in the completeness and timeliness of surveillance data at the end of the training program (16). The frontline FETP evaluation in Guinea showed that approximately 76% of the supervisors displayed that there were improvements in reporting specifically regarding its time and completeness. Also, 49% illustrated that they observed improvements in data analysis and data quality. About 97% of the supervisors have confirmed that case report analysis was done by the graduates routinely. About 96% of the graduates stated that the analysis of data had helped them in the early identification of outbreaks, following trends of reported diseases, starting investigations, and utilizing the surveillance data to come up with recommendations that would positively impact the surveillance procedures and public health (15).

The technical advisors in the advanced FETP evaluation in EMR reported that after establishing FETPs in the country, the data collection for the notifiable diseases had shown marked progress, as 70% of them reported an enhancement in the graduate's outbreak response and investigation, and all of them reported that graduates made a substantial contribution in enhancing the surveillance system. However, half of them reported that graduates were involved in presenting surveillance data. Also, regarding the FETP graduate's participation in the implementation and planning of interventions related to public health or evaluation, only 40% of the advisors reported that they were involved. Moreover, only 40% of the advisors confirmed that the FETP graduates play a crucial role in outbreaks (8).

It was acknowledged by the Ministry of Health and the graduates that the FELTP plays a crucial role in enhancing and strengthening the epidemiology curriculum. GFELTP graduates are deployed in strategic posts in the national public health service due to the presence of a policy by the Veterinary Services Directorate and the Ministry of Health, this demonstrates the graduate's abilities and skills (20).

On the other hand, the impact of FETP frontline on the data consistency was reported as minimal. Also, DCA (data consistency assessment) scores showed an 11.4% improvement between starting the training and a year and a half later (19).

#### Other outcomes

Five studies provided insights into other outcomes (9, 16–18, 20). In a study that evaluated the intermediate FETP in Tanzania, the majority of the trainees expressed favorable changes in their worksites, as they also experienced an increased sense of competencies to conduct audits, (summarize data for IDSR, analyze data and outbreak investigations), and assisting colleagues in these activities. It was noted by stakeholders that the intermediate FETP established a network of skillful epidemiologists that can recruited to enhance the data system and other outbreaks (16). Also, the advanced FETP evaluation study in India stated that the majority of the graduates reflected that the FETP advanced their careers (9). Additionally, in the UK advanced FETP evaluation study, it was stated that 15/16 of the graduates agreed or strongly agreed with the fact that the program resulted in advantages for their organization (17).

FETP had a positive impact on peer-reviewed scientific journals, manuscripts, and presentations (9, 18). Also, it was mentioned in India's advanced FETP evaluation that the publications of outbreak investigations that included malaria, typhoid, hepatitis E, cholera, and measles showed the use of epidemiological data for making decisions in the public health context (9). In addition to that, residents did oral and poster presentations that have been presented in global and regional scientific conferences (20).

The UK advanced FETP evaluation affirmed that FETP was found to have an impact on the capabilities, as results pointed out that the program enhanced the quality and capabilities of the national field epidemiology provision. Indirect impacts on the behaviors and the skills were noticed in the field epidemiology workforce and not only the new staff, which fostered networking within the field and implemented new practices and service enhancements. The interviews conveyed how and why participants sensed change brought by the FETP regarding their involvement and their preparedness levels. FETP graduates’ capabilities were influenced as they were seen as decision-makers, as they were able to cooperate with other organizations, and they were knowledgeable, as they also had wider insights (17). In India's advanced FETP evaluation, it was stated that FETP was found to help trainees and graduates in establishing connections (9).

#### Identified program gaps

The program gaps identified in each study varied depending on the country in which the FETP is being provided. The program gaps were mentioned in six studies (12, 13, 15, 16, 18, 20). The advanced FETP evaluation study in Yemen reported that the program doesn't cover all governorates and depends only on donor support which impacts the sustainability of the program (12). In the frontline FETP evaluation study in Ethiopia (13), gaps included a shortage of budget, mentors having a limited time for mentoring, lack of transportation, staff turnover, political instability, shortage of medical equipment and supplies which includes computers and internet, lack of career and professional development. The frontline FETP evaluation in Guinea reported some gaps such as gender imbalance and the recruiting senior trainees who are approaching retirement age (15). In the intermediate FETP evaluation study in Tanzania, program gaps included poor availability of mentors in the field assignments, difficulties in balancing the work responsibilities and the field assignments, and difficulties in accessing the data (16).

The advanced FETP evaluation in South Africa showed that the program didn't entice veterinary and medical graduates to enroll in the program which limits the number of people enrolled (18). This was similarly mentioned in the Ghana advanced FETP evaluation as it was stated that a limited number of qualified residents have been admitted to the program (20).

## Discussion

The review of FETP evaluations revealed encouraging findings regarding its influence on the skills and knowledge acquired by trainees and graduates, their active involvement in FETP activities, and enhancements in field epidemiological functions.

The five studies that evaluated the frontline FETP (7, 13–15, 19) and the two studies that advanced FETP (8, 9) reported a favorable impact on trainees’ knowledge and skills. The demonstrated impact of frontline FETPs remains consistent, whether it's based on self-reported assessments by trainees or favorable feedback from program implementers. Specifically, these evaluations noted enhanced skills and knowledge, particularly in surveillance activities, leading to improvements in data quality, early outbreak detection, rapid response, reporting, communication, and the guidance provided to surveillance and health facility workers (13).

These collective findings strongly emphasize the effectiveness and strength of the frontline FETP, despite its shorter duration, in enhancing the skills and knowledge of graduates.

The engagement of FETP graduates in Field epidemiology activities has been explored in just three studies that specifically assessed the advanced FETP (8, 18, 20), surprisingly neglecting this aspect in evaluations of the frontline FETP. The involvement of FETP graduates in field epidemiology is a pivotal evaluation indicator for frontline FETP. Their active engagement in outbreak investigations and surveillance activities stands as the primary outcome of these programs. Consequently, there is a critical need for additional data concerning the participation of graduates from frontline FETP in these crucial activities. Gathering more information about the involvement of these graduates will provide a clearer picture of the program's impact and effectiveness in real-world epidemiological practices.

While conducting outbreak investigations is expected from intermediate and advanced FETP graduates, the advanced program emphasizes using analytic epidemiology to lead or conduct such investigations (21). This emphasis becomes evident in the outcomes observed in the South African FETP, where residents actively participated and integrated themselves into the epidemiology and communicable diseases sections of health departments. SAFETP residents were instrumental in addressing various outbreaks across the African region. Their involvement extended to designing, implementing, and evaluating surveillance systems, significantly strengthening the public health surveillance network. Additionally, their role was pivotal in addressing and investigating multiple health concerns, particularly disease outbreaks, essential for informed public health decision-making (18). Similarly, findings from a study conducted in Jordan echo this sentiment, highlighting the graduates’ and residents’ swift identification of outbreaks, coupled with their ability to collect, analyze, and interpret data crucial for effective responses to disease outbreaks (8).

The evaluations of FETP programs have demonstrated substantial enhancements in epidemiological functions, particularly in data quality, analysis, reporting, and their impact on surveillance systems. However, the degree of improvement in these skills varies across different studies. For instance, one study evaluating the frontline FETP highlighted notable improvements in data analysis and reporting (15). In contrast, evaluations of intermediate FETP showcased marked enhancements in data quality and reporting practices (16). Both intermediate and frontline evaluations shed light on improvements in the completeness and timeliness of surveillance reports (15, 16).

In the case of the advanced FETP assessment, graduates actively engaged in supervising surveillance systems and analyzing surveillance data. Notably, 70% of technical advisors in one of the studies reported a noticeable enhancement in graduates’ outbreak response and investigation skills, with unanimous agreement on graduates significantly contributed to enhancing surveillance systems (8). This finding resonates with prior studies, which also indicated an increased number of trained field epidemiologists effectively performing core public health functions (22–24).

However, minimal impact has been observed concerning improvements in data consistency, notably highlighted in the evaluation of the frontline FETP (19). This observation is somewhat expected as frontline FETP graduates are primarily trained in interpreting and summarizing surveillance data. Nevertheless, achieving data consistency is crucial for ensuring accurate and coherent results, ultimately facilitating valuable assessments, informed analysis, decision-making, and comprehensive reporting (24).

One crucial aspect examined in FETP evaluations is scientific communication (21). Graduates from different levels of FETP have varying expectations in this domain. Advanced FETP graduates are anticipated to develop comprehensive oral and written epidemiological reports for external audiences, while intermediate FETP graduates should produce and disseminate epidemiologic reports externally. Frontline FETP graduates, on the other hand, are expected to generate timely surveillance reports for internal use (21).

Advanced FETP graduates demonstrated proficiency in preparing scientific manuscripts for journals and delivering presentations (9, 18). This highlights the program's positive influence on scientific communication among advanced FETP graduates. Additionally, evaluations indicated that intermediate FETP graduates exhibited increased competencies in conducting audits (16).

The observed positive impact on scientific communication aligns with findings from the Epidemic Intelligence Service (EIS) training program, showcasing its influence on enhancing scientific literature and fortifying public health infrastructure at both state and local levels (25). These findings collectively underscore the program's effectiveness in nurturing effective scientific communication skills across advanced and intermediate FETP training.

The majority of the evaluation studies focused solely on one group—trainees enrolled in the FETP. However, a more robust approach was observed in only two studies, which involved comparing two groups: the trained individuals vs. an untrained group (13, 14). Research methodologies involving two groups offer significant advantages over single-group studies. They enable direct comparisons, facilitating a clearer understanding of the program's impact, especially in discerning differences. Moreover, this approach enhances internal validity and mitigates the influence of confounding variables, thereby reducing bias.

For instance, a study by Kebebew et al. (14) demonstrated this by contrasting the activities and outcomes between FETP-trained individuals and an untrained (control) group. This comparison delineated the percentage differences observed, effectively showcasing the FETP's impact on trained individuals in contrast to those unexposed to the program. This controlled comparison highlights the specific influence of FETP training on the students, isolating it from other external factors that might affect their competencies.

However, the limitation in most studies lies in their evaluation of the FETP's impact solely on the trained students or graduates, lacking a control group of untrained individuals for comparison. This absence makes it challenging to unequivocally attribute the observed competencies solely to FETP training. Incorporating control groups into future studies would greatly enhance the ability to discern and attribute the specific impact of FETP training on the skills and knowledge acquired by participants.

In one study, PHEP-SPO and PHEP nutrition programs were highlighted (7). PHEP-SPO focuses on equipping individuals with skills crucial for sustaining polio eradication, promoting health empowerment, and bolstering immunization systems. On the other hand, PHEP nutrition primarily aims to enhance knowledge and skills to contribute effectively to nutrition and related interventions, particularly in child and maternal health.

Interestingly, the results showcased that graduates from these two programs reported lower perceived improvements in their abilities related to field epidemiology activities when compared to those who completed the PHEP-BFE, the basic field epidemiology program. This suggests the need for a comprehensive review to optimize the outcomes of these programs. A thorough assessment of the curriculum is essential to identify areas for improvement that can positively impact the outcomes (26). This revision could potentially enhance the capabilities and contributions of graduates from the PHEP-SPO and PHEP nutrition programs in the field of epidemiology.

Engaging diverse stakeholders in FETP evaluation research is critical for conducting comprehensive and relevant evaluation studies. Involving multiple viewpoints enriches the perspective and transforms research findings into actionable strategies. This multifaceted approach not only enhances understanding but also facilitates tailored interventions to address specific needs. Furthermore, it bolsters research validity by mitigating bias and amplifies the potential impact of research outcomes (27).

Therefore, FETP evaluation studies should encompass input from various stakeholders, including public health practitioners, program participants, and graduates, to glean insights into their experiences and the program's impact on their skills and knowledge. Program coordinators’ perspectives are invaluable for understanding curriculum development, training activities, and program assessment. Mentors and supervisors contribute essential data on how FETP influences trainees during field assignments. Involving data providers, users, and community representatives engaged in FETP, as well as government and non-governmental organizations, helps capture diverse perspectives, collaborations, and support for field investigations and resources provided.

For instance, an advanced FETP evaluation conducted in Yemen (12) engaged a broad spectrum of stakeholders, including technical staff, overseeing policymakers, program directors, employing organizations, and graduates. This extensive inclusion of stakeholders resulted in richer, more comprehensive insights, enhancing the validity and relevance of the outcomes. The research underscores that stakeholder engagement enriches both the theoretical robustness and practical applicability of research. Some studies have highlighted significant program gaps that require attention. Firstly, the lack of national coverage across all governorates and heavy reliance solely on donor support significantly impacts the program's sustainability (12). To address this issue, diversifying funding sources, establishing partnerships, and conducting thorough needs assessments to identify training demands and key areas requiring coverage are essential steps (28). Creating a comprehensive expansion strategy prioritizing areas based on critical needs can help achieve wider coverage.

Additionally, identified gaps such as budget shortages, inadequate medical equipment and supplies, and a deficit in human resources can be mitigated through strategic partnerships for financial support, targeted training for existing personnel, and skills development initiatives (13). Enhancing mentorship quality can be achieved through specialized training programs for mentors, improving their skills and effectiveness. To combat the lack of career development opportunities, implementing programs focusing on professional growth, conducting seminars, and workshops can bolster knowledge and skills. Implementing a clearly defined mentor availability timetable can address the issue of limited mentor support. Lastly, strategies aimed at providing career advancement pathways and professional development opportunities can help mitigate staff turnover.

Concerning gender imbalance and the focus on recruiting senior trainees nearing retirement age (15), evaluating policies to identify gender biases and rectifying any policies that don't provide equal opportunities for both genders is crucial. Establishing recruitment criteria that emphasize the importance of diverse age ranges and balanced experience levels can ensure a more inclusive and robust recruitment process.

In one study, it was noted that the program faced challenges in attracting veterinary and medical graduates, thereby limiting enrollment numbers (18). To address this issue, promoting the program through robust outreach efforts can enhance its visibility and attract more participants. Conducting a needs assessment to understand the reasons behind the low enrollment among graduates could provide valuable insights into the barriers and aid in devising effective solutions.

Additionally, another study highlighted the constraint of limited time for data analysis (16). This challenge can be mitigated by organizing time management workshops focused on effective strategies for allocating specific time to data analysis. Enhancing participants’ skills in prioritizing tasks, especially data analysis, can significantly improve efficiency.

Furthermore, the difficulty in balancing work responsibilities and field assignments could be addressed by meticulous scheduling, goal setting, and task prioritization. The limited flexibility in work schedules, which hampers data access and survey participant engagement for field assignments, suggests exploring hybrid models combining remote and onsite data collection. Such approaches not only enhance flexibility but also reduce travel costs associated with fieldwork. Each study identified several limitations within its scope. A common limitation across many studies was the reliance on self-assessment by individuals (8, 9, 13, 15, 16, 19). Additionally, studies highlighted potential bias stemming from favorable views possibly influenced by team members involved in the FETP project (12, 17), which might lead to either overestimation or underestimation of competencies or skills.

Moreover, some studies primarily focused on respondents’ program responses and challenges, neglecting in-depth exploration of themes and perceptions (13). Some evaluations also lacked consideration of the program's long-term impact, focusing solely on input, process, and output (9, 12). Another limitation observed in one study was the focus on participants’ involvement in field activities and their perceived skills, overlooking an evaluation of program competencies (8). Additionally, there was a lack of direct measures for evaluating the surveillance system in one study (15). Comparative studies between trained and untrained officers revealed differences between groups, including educational background and participation in other training programs (14).

One of the strengths of this study is its coverage of evaluations of FETPs across three different program modalities (frontline, intermediate, advanced) in various countries and regions, offering a diverse and comprehensive understanding of the programs’ global impacts. This cross-comparison, which is relatively rare in the literature, provides a broader perspective on the effectiveness and challenges of FETPs in different settings. Additionally, the review highlighted the methodological diversity in FETP evaluations, enriching the understanding of how FETPs are assessed and the various approaches used to measure their impact.

A limitation of this review is the limited attention given to intermediate FETP evaluations, with only one study considered, insufficient to comprehensively review the program's impact on competencies. This could be explained by the fact that intermediate FETP is relatively new and implemented in fewer countries compared to the advanced program.

## Conclusion

This review showed the substantial positive impact of FETPs on trainees and graduates, highlighting significant competency enhancements across different program modalities. The findings demonstrate notable improvements in skills and knowledge, active engagement in FETP activities, and advancements in field epidemiological functions. These outcomes underscore the critical role of FETPs in building a proficient public health workforce. The review also revealed specific strengths and gaps within FETP implementations across various regions. While many graduates reported enhanced skills in surveillance and outbreak response, challenges such as budget constraints, mentor availability, and resource limitations were prevalent. Addressing these gaps is essential for maximizing the effectiveness and sustainability of FETPs. The evaluation of FETPs is an ongoing and collaborative process, requiring concerted efforts from program administrators, graduates, stakeholders, and the broader public health community. To further improve FETP evaluations, evaluators need to adopt diverse and robust frameworks, such as Kirkpatrick's model, to comprehensively assess FETP outcomes at multiple levels. Several strategies are recommended to enhance FETP evaluations such as establishing clear and measurable objectives aligned with the program's mission and goals, developing robust evaluation tools and standardized metrics to compare FETPs across regions or countries, enabling benchmarking and identification of areas needing improvement, gathering feedback from stakeholders, including health ministries, public health agencies, and communities served by FETP graduates, to refine the training, and implementing longitudinal tracking systems to monitor the progress of FETP graduates, follow up on their careers, and assess their contributions to public health and application of learned skills.

## Data Availability

The datasets presented in this study can be found in online repositories. The names of the repository/repositories and accession number(s) can be found in the article/supplementary material.
